# Greater corneal nerve loss at the inferior whorl is related to the presence of diabetic neuropathy and painful diabetic neuropathy

**DOI:** 10.1038/s41598-018-21643-z

**Published:** 2018-02-19

**Authors:** Alise Kalteniece, Maryam Ferdousi, Ioannis Petropoulos, Shazli Azmi, Safwaan Adam, Hassan Fadavi, Andrew Marshall, Andrew J. M. Boulton, Nathan Efron, Catharina G. Faber, Giuseppe Lauria, Handrean Soran, Rayaz A. Malik

**Affiliations:** 1 Institute of Cardiovascular Sciences, Cardiac Centre, Faculty of Medical and Human Sciences, University of Manchester and NIHR/Wellcome Trust Clinical Research Facility, Manchester, UK; 2 Weill Cornell Medicine-Qatar, Research Division, Qatar Foundation, Education City, Doha, Qatar; 3 Queensland University of Technology, School of Optometry and Vision Science, Brisbane, Australia; 4 Department of Neurology, School of Mental Health and Neuroscience, Maastricht University Medical Center, Maastricht, The Netherlands; 5 Neuroalgology Unit, IRCCS Foundation, “Carlo Besta” Neurological Institute, Milan, Italy; 6 Department of Biomedical and Clinical Sciences, “Luigi Sacco”, University of Milan, Milan, Italy

## Abstract

We assessed whether a measure of more distal corneal nerve fibre loss at the inferior whorl(IW) region is better than proximal measures of central corneal nerve damage in relation to the diagnosis of diabetic peripheral neuropathy(DPN), painful DPN and quality of life(QoL). Participants underwent detailed assessment of neuropathy, QoL using the SF36 questionnaire, pain visual analogue score(VAS), and corneal confocal microscopy(CCM). Corneal nerve fibre density (CNFD), branch density (CNBD) and length (CNFL) at the central cornea and inferior whorl length (IWL) and average(ANFL) and total(TNFL) nerve fibre length were compared in patients with and without DPN and between patients with and without painful DPN and in relation to QoL. All CCM parameters were significantly reduced, but IWL was reduced ~three-fold greater than CNFL in patients with and without DPN compared to controls. IWL(p = 0.001), ANFL(p = 0.01) and TNFL(p = 0.02) were significantly lower in patients with painful compared to painless DPN. The VAS score correlated with IWL(r = −0.36, P = 0.004), ANFL(r = −0.32, P = 0.01) and TNFL(r = −0.32, P = 0.01) and QoL correlated with CNFL(r = 0.35, P = 0.01) and IWL(r = 0.4, P = 0.004). Corneal nerve fibre damage is more prominent at the IW, lower in patients with painful compared to painless neuropathy and relates to their QoL. IWL may provide additional clinical utility for CCM in patients with DPN.

## Introduction

Diabetic peripheral neuropathy (DPN) affects up to 50% of all patients with diabetes^1^. It is the dominant cause of increased morbidity through painful neuropathy and foot ulceration and is also associated with increased mortality^2–4^. In 2003, we demonstrated the potential of corneal confocal microscopy (CCM) as a rapid and non-invasive means to quantify central corneal nerve morphology in early and more advanced DPN^5^. Subsequently, many studies have confirmed the utility of CCM in quantifying nerve fibre damage in diabetic neuropathy^3,6–9^.

We and others have also reported corneal nerve fibre loss at the more distal inferior whorl region of the cornea in patients with DPN^10,11^ and progressively greater nerve fibre damage when moving from the central cornea to the inferior whorl and corneal epithelium in animal models of DPN^12^. This is consistent with a dying back neuropathy affecting the more distal nerve fibres in DPN. Additionally, the inferior whorl (IW) is a distinctive spiralled, clockwise sub-basal nerve fibre pattern located in the inferonasal cornea^13,14^. Because of its unique pattern, it has been suggested that it may be a more reliable landmark for longitudinal and interventional assessment of the corneal sub-basal nerve plexus^15^. Two previous reports showed that corneal nerve fibre length at the IW and centre has comparable sensitivity and reliability for detecting DPN^11,16^. We have also previously suggested that a combination of corneal nerve fibre length at the centre and inferior whorl may increase the sensitivity of CCM in detecting DPN^11^.

Painful neuropathy affects approximately 21% of patients with diabetes^17,18^ and can significantly reduce the quality of life^19,20^. A small study has demonstrated a greater reduction in central corneal nerve fibre length in patients with painful diabetic neuropathy^3^. In the present study, we have compared corneal nerve pathology in the central and inferior whorl regions in relation to the severity and presence of DPN and painful DPN and quality of life.

## Methodology

### Study subjects

116 patients with type 1 or type 2 diabetes and 22 age-matched healthy controls underwent assessment of peripheral neuropathy and corneal confocal microscopy (CCM). Patients were excluded if they had a history of malignancy, neuropathy of non-diabetic cause, current or active diabetic foot ulceration, deficiency of B12 or folate, chronic renal impairment or liver failure, connective tissue or systemic disease, corneal trauma, systemic disease that involves the cornea and cystic corneal disorders. Before participation informed consent was obtained from each participant. This research adhered to the tenets of Declaration of Helsinki and was approved by Greater Manchester east Research Ethics Committee.

### Clinical and peripheral neuropathy assessment

All participants underwent assessment of body mass index (BMI) and blood pressure. Neurological deficits were assessed using the Neuropathy Disability Score (NDS), which includes an assessment of vibration perception, pinprick, temperature sensation and presence or absence of ankle reflexes^21^. Based on the NDS score patients were divided into two groups: without neuropathy (DN−) (NDS < 3) (n = 47) and with neuropathy (DN+) (NDS ≥ 3) (n = 69). VPT was assessed using a Horwell Neurothesiometer (Scientific Laboratory Supplies, Wilfrod, Nottingham, UK). Cold (CT) and warm (WT) perception thresholds, were assessed on the dorsolateral aspect of the non-dominant foot (S1) using a TSA-II NeuroSensory Analyser (Medoc, Ltd., Ramat-Yishai, Israel). Electrodiagnostic studies were undertaken using a Dantec ‘’Keypoint” system (Dantec Dynamics Ltd, Bristol, UK), equipped with a DISA temperature regulator to keep the limb temperature constantly at 32–35 °C. Sural sensory nerve amplitude (SSNA), sural sensory nerve conduction velocity (SSNCV), peroneal motor nerve amplitude (PMNA), and peroneal motor nerve conduction velocity (PMNCV) were assessed by a consultant neurophysiologist.

Quality of life was assessed using The Short Form 36 Health Survey (SF-36) questionnaire [15]. This survey consists of 36 questions, measures eight different dimensions, which are scored from 0 to 100. We used SF-36 total score, an average of eight dimensions as a single measure of quality of life.

A diagnosis of painful diabetic neuropathy was based on the presence of typical symmetrical neuropathic symptoms such as burning, aching pain in the feet and a visual analogue scale (VAS) > 4 mm in patients with abnormal distal sensation or decreased/absent distal reflexes (NDS ≥ 3)^22^.

### Corneal Confocal Microscopy

All participants underwent CCM examination using a laser scanning corneal confocal microscope HRT III (Heidelberg Retinal Tomograph III Rostock Cornea Module, Heidelberg Engineering, Heidelberg, Germany) for both eyes using our established protocol for the central and inferior whorl area of the cornea^11,23^. Six images from the central cornea and four images from inferior whorl at the level of sub-basal nerve plexus were selected and manually quantified using CCMetrics (The University of Manchester, Manchester, UK). Images were selected based on their quality and variability using our established protocol^11,24^. Four corneal parameters were quantified: corneal nerve fibre density (CNFD – total number of main nerves per square millimetre (no./mm^2^), corneal nerve branch density (CNBD – number of nerve branches per square millimetre), corneal nerve fibre length (CNFL – total length of main nerves and nerve branches per square millimetre) (mm/mm^2^), inferior whorl length (IWL – total length of nerves per square millimetre at the IW region) (mm/mm^2^), average nerve fibre length (ANFL) − (CNFL + IWL/2 (mm/mm^2^)) and total nerve fibre length (TNFL) − (CNFL + IWL (mm/mm^2^)).

### Statistical analysis

Analysis was carried out using SPSS (Version 22.0 for Macintosh, IBM Corporation, New York, NY, USA). Independent T-test (Mann Witney U test for nonparametric variables) was used to assess the estimates between two groups. The One-Way Anova (post hoc *LSD*) was used to compare means among groups. The analysis of covariance (Ancova) (post hoc LSD) was used to compare variables between groups, while statistically controlling for the effects of age. *Pearson’s* correlation coefficient (Spearman’s for non-parametric) was calculated to assess the associations among different variables. All the data are expressed as mean ± standard error (SE). P < 0.05 was considered as significant. Graphpad Prism (Version 7.0c for Macintosh, Graphpad Software, La Jolla California, USA) was used for plotting the graphs.

### Data availability

The datasets generated during and/or analysed during the current study are available from the corresponding author on reasonable request.

## Results

### Clinical assessment

There was no significant difference in age between control subjects and patients with diabetes (50.32 ± 2.92 vs. 55.97 ± 1.32, P = 0.08), but patients with DN+ were older (50.32 ± 2.92 vs. 62.08 ± 1.4, P = 0.001). Therefore, the values were adjusted for age using analysis of covariance (ANCOVA). BMI (kg/m^2^) (26.47 ± 1.15 vs. 29.58 ± 0.73, P = 0.029) and HbA1c (mmol/mol) (36.4 ± 1.02 (5.48%) vs. 57.49 ± 1.66 (7.41%), P < 0.0001) were higher and total cholesterol (mmol/L) (5.27 ± 0.17 vs. 4.26 ± 0.11, P < 0.0001) was lower in diabetic patients compared to control subjects (Table 1). Systolic BP (mmHg) (135.60 ± 2.08 vs. 126.58 ± 2.23, P = 0.02) and BMI (kg/m^2^) (31.29 ± 1.04 vs. 27.49 ± 0.93, P = 0.02) were significantly higher in DN+ compared to DN−.

**Table 1:** Demographic and neuropathy measures in patients with diabetes compared to controls.

	Controls (n = 22)	Diabetes (n = 116)	P value
Age	50.32 ± 2.92	55.97 ± 1.32	0.088
Ethnicity (European/Asian/African)	18/3/1	90/20/3	
Gender (female/men)	11/11	49/67	0.6
Duration of diabetes	N/A	18.85 ± 1.47	
Type of diabetes (T1DM/T2DM)	N/A	52/63	
BP Sys (mmHg)	125.95 ± 4.47	131.83 ± 1.58	0.226
Total cholesterol (mmol/L)	5.27 ± 0.17	4.26 ± 0.11	<0.0001
BMI (kg/m^2^)	26.47 ± 1.15	29.58 ± 0.73	0.029
HbA1c (mmol/mol)	36.4 ± 1.02	57.49 ± 1.66	<0.0001
HbA1c (%)	5.48 ± 0.09	7.41 ± 0.15	<0.0001
NCCA (mbars)	0.66 ± 0.10	0.85 ± 0.09	0.192
CNFD (no./mm^2^)	36.33 ± 1.49	25.14 ± 0.77	<0.0001
CNBD (no./mm^2^)	90.84 ± 7.41	60.14 ± 2.99	0.001
CNFL (mm/mm^2^)	26.76 ± 1.04	22.39 ± 0.65	0.001
IWL (mm/mm^2^)	35.31 ± 2.11	22.73 ± 0.88	<0.0001
ANFL (mm/mm^2^)	31.03 ± 1.22	22.56 ± 0.68	<0.0001
TNFL (mm/mm^2^)	62.07 ± 2.45	45.12 ± 1.36	<0.0001
PMNCV (m/s)	47.52 ± 1.05	42.27 ± 0.46	<0.0001
SSNCV (m/s)	50.41 ± 1.24	43.18 ± 0.66	<0.0001
PMNA (mV)	5.14 ± 0.36	3.82 ± 0.29	0.006
SSNA (μV)	17.61 ± 1.74	10.26 ± 0.72	0.001
CT (°C)	12.30 ± 2.13	20.61 ± 0.94	0.001
WT (°C)	45.78 ± 0.66	42.68 ± 0.55	0.001
VPT (V)	6.53 ± 1.1	14.79 ± 0.93	<0.0001

Results are expressed as Mean ± SE.

### Neuropathy assessment

PMNCV (m/s) (43.42 ± 0.78 vs. 47.52 ± 1.05, P = 0.004) and SSNCV (m/s) (44.39 ± 1.36 vs. 50.41 ± 1.24, P = 0.003) were significantly lower and CT (°C) (19.71 ± 1.46 vs. 12.30 ± 2.13, P = 0.016) was significantly higher in DN− compared to control subjects. PMNCV (m/s) (41.48 ± 0.54 vs. 47.52 ± 1.05, P < 0.0001), SSNCV (m/s) (42.36 ± 0.60 vs. 50.41 ± 1.24, P < 0.0001), PMNA (mV) (3.14 ± 0.23 vs. 5.14 ± 0.36, P = 0.014) and SSNA (μV) (8.47 ± 0.90 vs. 17.61 ± 1.74, P < 0.0001) were significantly lower and VPT (V) (19.09 ± 1.22 vs. 6.53 ± 1.1, P < 0.0001) and CT (°C) (21.21 ± 1.03 vs. 12.30 ± 2.13, P = 0.001) were significantly higher in patients with DN+ compared to controls. PMNA (mV) (4.76 ± 0.59 vs. 3.14 ± 0.23, P = 0.009) and SSNA (μV) (12.86 ± 1.11 vs. 8.47 ± 0.90, P = 0.01) were lower and VPT (V) was higher (19.09 ± 1.22 vs. 8.29 ± 0.69, P < 0.0001) in DN+ compared to DN− (Table 2).

**Table 2:** Demographic and neuropathy status of healthy controls, patients without (DN−) and with (DN+) diabetic neuropathy.

	Controls (n = 22)	DN − (n = 47)	DN+ (n = 69)
Age	50.32 ± 2.92	46.90 ± 1.93^§^	62.08 ± 1.4^^^
Ethnicity (European/Asian/African)	18/3/1	38/6/1	52/14/2
Gender (female/men)	11/11	21/25	28/41
Duration of diabetes	N/A	16.04 ± 1.78	20.78 ± 2.14
Type of diabetes (T1DM/T2DM)	N/A	29/17	23/46
BP Sys (mmHg)	125.95 ± 4.47	126.58 ± 2.23^$^	135.60 ± 2.08
Cholesterol (mmol/l)	5.27 ± 0.17	4.34 ± 0.17*	4.19 ± 0.15^^^
BMI (kg/m^2^)	26.47 ± 1.15	27.49 ± 0.93^$^	31.29 ± 1.04*
HbA1c (mmol/mol)	36.4 ± 1.02	60.88 ± 3.32^^^	55.19 ± 11.95^^^
HbA1c (%)	5.48 ± 0.09	7.72 ± 0.30*	7.19 ± 0.14^^^
CNFD (no./mm^2^)^£^	35.42 ± 1.6	26.31 ± 1.19*	24.5 ± 0.9^^^
CNBD (no./mm^2^)^£^	89.64 ± 7.25	64.29 ± 5.12^^^	56.94 ± 4.11^^^
CNFL (mm/mm^2^)^£^	26.57 ± 1.45	23.16 ± 1.06*	21.84 ± 0.87^^^
IWL (mm/mm^2^)^£^	35.18 ± 2.02	24.08 ± 1.4*	21.56 ± 1.2^^^
ANFL (mm/mm^2^)^£^	30.88 ± 1.51	23.62 ± 1.1^⊥^	21.70 ± 0.9^^^
TNFL (mm/mm^2^)^£^	61.75 ± 3.02	47.24 ± 2.21^^^	43.4 ± 1.8^^^
PMNCV (m/s)	47.52 ± 1.05	43.42 ± 0.78^^^	41.48 ± 0.54^^^
SSNCV (m/s)	50.41 ± 1.24	44.39 ± 1.36^^^	42.36 ± 0.60^^^
PMNA (mV)	5.14 ± 0.36	4.76 ± 0.59^$^	3.14 ± 0.23*
SSNA (μV)	17.61 ± 1.74	12.86 ± 1.11^§^	8.47 ± 0.90^^^
CT (°C)	12.30 ± 2.13	19.71 ± 1.46*	21.21 ± 1.03^^^
WT (°C)	45.78 ± 0.66	42.29 ± 0.69	42.94 ± 0.8
VPT (V)	6.53 ± 1.1	8.29 ± 0.69^§^	19.09 ± 1.22^^^
SF-36 (0–100)^£^	N/A	69.12 ± 6.4	58.43 ± 3.7

^£^Represents values adjusted for age using ANCOVA. *P < 0.05 compared to controls, ^$^P < 0.05 compared to DN+, P < 0.01 compared to controls, ^§^P < 0.01 compared to DN+.

The total SF-36 score was reduced in DN− (69.12 ± 6.4) and DN+ (58.43 ± 3.7) but did not differ between the two (P = 0.1) (Table 2).

### Corneal Confocal Microscopy

**Figure 1:**
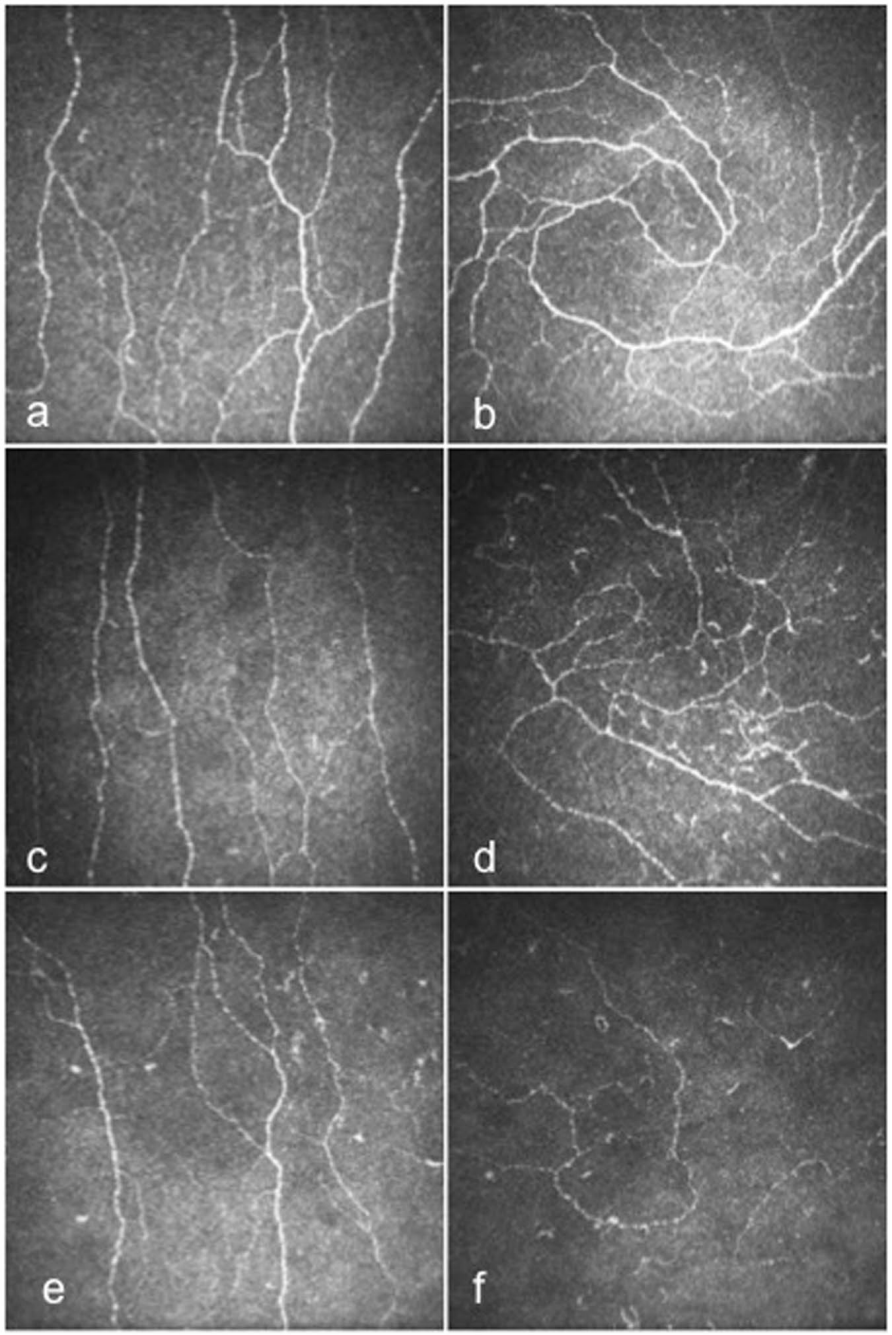
CCM images of the central cornea and IW in control subject (**a** and **b**), in a patient with DN− (**c** and **d**) and in a patient with DN+ (**e** and **f**).

**Figure 2:**
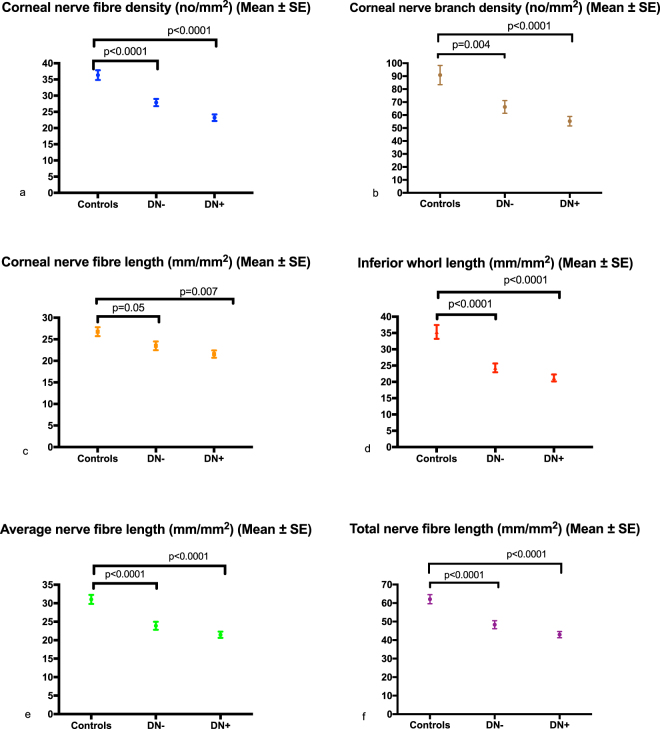
Error bar plots for corneal nerve fibre density (CNFD) (**a**), corneal nerve branch density (CNBD) (**b**), corneal nerve fibre length (CNFL) (**c**), Inferior whorl length (IWL) (**d**), average nerve fibre length (ANFL) (**e**) and total nerve fibre length (TNFL) (**f**) in controls and diabetic patients without (DN−) and with (DN+) neuropathy.

Corneal nerve fibre density (no./mm^2^) (25.14 ± 0.77 vs. 36.33 ± 1.49, p < 0.0001), branch density (no./mm^2^) (60.14 ± 2.99 vs. 90.84 ± 7.41, P = 0.001), length (mm/mm^2^) (22.39 ± 0.65 vs. 26.76 ± 1.04, p = 0.001, −16.3%), inferior whorl length (mm/mm^2^) (22.73 ± 0.88 vs. 35.31 ± 2.11, p < 0.0001, −35.6%), average (mm/mm^2^) (22.56 ± 0.68 vs. 31.03 ± 1.22, P < 0.0001, −27.3%) and total (mm/mm^2^) (45.12 ± 1.36 vs. 62.07 ± 2.45, P < 0.0001, −27.3%) nerve fibre length were significantly lower in patients with diabetes compared to controls (Table 1). Corneal nerve fibre density (no./mm^2^) (26.31 ± 1.19 vs. 35.42 ± 1.6, P < 0.0001), branch density (no./mm^2^) (64.29 ± 5.12 vs. 89.64 ± 7.25, P = 0.004), length (mm/mm^2^) (23.16 ± 1.06 vs. 26.57 ± 1.45, P = 0.05, −12.8%), inferior whorl length (mm/mm^2^) (24.08 ± 1.4 vs. 35.18 ± 2.02, P < 0.0001, −31.6%), average (mm/mm^2^) (23.62 ± 1.1 vs. 30.88 ± 1.51, P < 0.0001, −23.5%) and total (mm/mm^2^) (47.24 ± 2.21 vs. 61.75 ± 3.02, P < 0.0001, −23.5%) nerve fibre length were significantly lower in DN− compared to controls, after adjustment for age. Corneal nerve fibre density (no./mm^2^) (24.5 ± 0.9 vs. 35.42 ± 1.6, P < 0.0001), branch density (no./mm^2^) (56.94 ± 4.11 vs. 89.64 ± 7.25, P < 0.0001), length (mm/mm^2^) (21.84 ± 0.87 vs. 26.57 ± 1.45, P = 0.007, −17.8%), inferior whorl length (mm/mm^2^) (21.56 ± 1.2 vs. 35.18 ± 2.02, P < 0.0001, −38.7%), average (mm/mm^2^) (21.70 ± 0.9 vs. 30.88 ± 1.51, P < 0.0001, −29.7%) and total (mm/mm^2^) (43.4 ± 1.8 vs. 61.75 ± 3.02, P < 0.0001, −29.7%) nerve fibre length were significantly reduced in patients with DN+ compared to controls, after adjustment for age (Figs 1 and 2). There was no significant difference in any CCM parameter between diabetic patients with and without DPN (Table 2).

**Figure 3:**
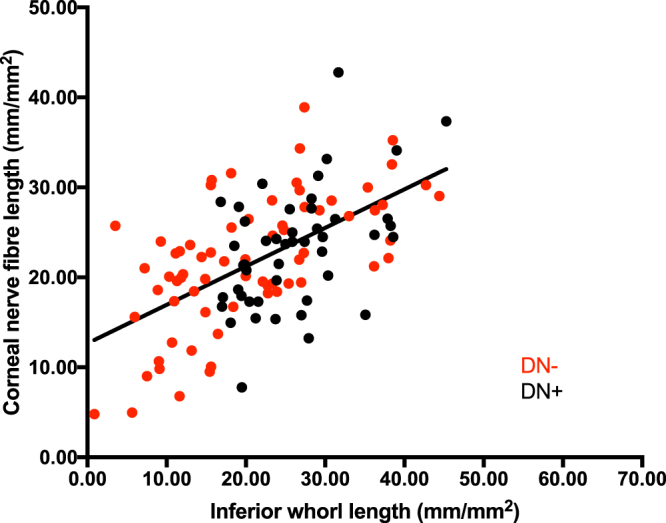
The association between inferior whorl length (IWL) and corneal nerve fibre length (CNFL).

The Mean-2SD of controls was used to define an abnormal corneal nerve fibre length and IWL. 30% of patients with diabetes had damage in the inferior whorl but normal corneal nerve fibre length, whereas 13.5% of patients had damage in corneal nerve fibre length but a normal inferior whorl length. The scatterplot (Fig. 3) shows the relationship between inferior whorl length and corneal nerve fibre length. Inferior whorl length correlated significantly with corneal nerve fibre length in patients without (ICC = 0.75, P < 0.0001) and with (ICC = 0.66, P < 0.0001) neuropathy.

Corneal nerve branch density (r = 0.3, P = 0.008), length (r = 0.35, P = 0.01), inferior whorl length (r = 0.4, P = 0.004), average (r = 0.5, P = 0.001) and total (r = 0.5, P = 0.001) nerve fibre length correlated significantly with the total SF-36 score. There was no significant difference in corneal nerve fibre density, branch density or length, but inferior whorl length (mm/mm^2^) (17.25 ± 1.49 vs. 24.59 ± 1.57, P = 0.001), average (mm/mm^2^) (18.86 ± 1.16 vs. 23.38 ± 1.24, P = 0.01) and total (mm/mm^2^) (38.28 ± 2.5 vs. 46.54 ± 2.34, P = 0.02) nerve fibre length, were significantly lower in patients with painful compared to painless diabetic neuropathy (Table 3). There was a significant correlation between the severity of pain (VAS score) with inferior whorl length (r = −0.36, P = 0.004), average (r = −0.32, P = 0.01) and total (r = −0.32, P = 0.01) nerve fibre length. There was no significant difference for any CCM parameter in patients with Type 1 and Type 2 diabetes after adjusting for age, duration of diabetes and HbA1c (Supplementary Table S1).

**Table 3:** CCM parameters in controls, patients with painless and painful neuropathy.

	Controls (n = 22)	Painless neuropathy (n = 33)	Painful neuropathy (n = 27)
Age	50.32 ± 2.92	59.97 ± 2.12^^^	64.55 ± 2.2^^^
Duration of diabetes	N/A	23.69 ± 3.31	18.11 ± 3.02
CNFD (no./mm^2^)^£^	36.33 ± 1.49	25.01 ± 1.45^^^	21.16 ± 1.58^^^
CNBD (no./mm^2^)^£^	86.97 ± 6.9	58.93 ± 5.07^^^	50.44 ± 5.5^^^
CNFL (mm/mm^2^)^£^	26.76 ± 1.04	22.17 ± 1.24*	20.48 ± 1.31^^^
IWL (mm/mm^2^)^£^	35.31 ± 2.11	24.59 ± 1.57^^II^	17.25 ± 1.49^^^
ANFL (mm/mm^2^)^£^	31.03 ± 1.22	23.38 ± 1.24^^II^	18.86 ± 1.16^^^
TNFL (mm/mm^2^)^£^	60.75 ± 2.9	46.54 ± 2.34^^$^	38.28 ± 2.5^^^

^£^Represents values adjusted for age using ANCOVA; *P < 0.05 compared to controls, ^$^P < 0.05 compared to painful neuropathy; ^^^P < 0.01 compared to controls; ^II^P < 0.01 compared to painful neuropathy.

## Discussion

CCM is a rapid, reproducible ophthalmic technique that can quantify corneal nerve fibre abnormalities in diabetic and a number of other peripheral neuropathies^3,25–28^. Two recent studies have shown that CCM has a diagnostic ability, which is comparable to the current gold standard technique of skin biopsy^9,29^. We confirm that CCM can detect sub-clinical corneal nerve damage in patients without evidence of DPN^11^.

DPN is length-dependent neuropathy and will therefore affect the most distal nerve fibres first^30^ i.e. the nerves at the inferior whorl which are more distal to the nerves in the central cornea. Corneal nerve loss has been reported to be comparable at the inferior whorl and central cornea in diabetic patients with and without neuropathy^11,15,16^. However, in the present study, we demonstrate a ~2–3-fold greater reduction in corneal nerve fibre length at the IW compared to the central cornea in diabetic patients with and without DPN compared to controls. We also show that whilst 30% of patients with a reduction in inferior whorl length had a normal corneal nerve fibre length, only 13.5% of patients with a normal inferior whorl length had an abnormal corneal nerve fibre length; confirming more prominent distal corneal nerve loss. Given this difference we postulated that a combination of both corneal nerve fibre length and inferior whorl length, either as a total or average length may be more useful than either measure alone. Indeed, inferior whorl length, average and total nerve fibre length rather than the standard parameters of corneal nerve fibre density, branch density and length correlate with the quality of life and the presence and severity of painful diabetic neuropathy. Given that new therapies for diabetic neuropathy should induce nerve repair in the most distal part of the nerve, we propose that quantification of inferior whorl length and the average and total nerve fibre length may provide a more powerful means to assess a therapeutic response than has already been demonstrated by quantifying central corneal nerve morphology^31–33^. An additional advantage is the unique pattern of the IW, which facilitates its use as a landmark in longitudinal and interventional studies.

To our knowledge the association between CCM parameters and QoL and presence and severity of neuropathic pain has not been previously reported. QoL is an important patient outcome and we used the validated SF-36 questionnaire^34^ to assess the QoL in patients in this study. We show a more significant correlation between QoL and IWL compared to corneal nerve fibre length. Furthermore, inferior whorl length, average and total nerve fibre length differentiate patients with and without painful neuropathy and correlate with the severity of painful neuropathy, whereas corneal nerve fibre density, branch density and length, do not.

A potential limitation of our study is that the diagnosis of painful diabetic neuropathy was principally based on the VAS and NDS scores, but this is in accord with the IASP definition for neuropathic pain as adopted by a recent large painful neuropathy phenotyping study^20^. We believe future studies should better stratify cases in relation to the severity and sub-types of diabetic neuropathy.

In conclusion we have shown that there is more prominent distal corneal nerve fibre damage at the inferior whorl in patients with diabetic neuropathy. It also provides an objective measure of small nerve fibre damage, which relates to the presence of painful diabetic neuropathy and the quality of life. Further studies deploying these novel measures of more distal corneal nerve damage and repair are required in longitudinal studies and in clinical trials of new therapies to define their clinical utility.

## Electronic supplementary material

Supplementary table S1
