# Supplementary material for: Greater corneal nerve loss at the inferior whorl is related to the presence of diabetic neuropathy and painful diabetic neuropathy
Source: Sci Rep. 2018 Feb 19;8:3283. doi: 10.1038/s41598-018-21643-z (PMC5818543; doi:10.1038/s41598-018-21643-z)
Supplement: Supplementary file 1 — Supplementary table S1 [file 41598_2018_21643_MOESM1_ESM.pdf]

Greater corneal nerve loss at the inferior whorl is related to the presence of diabetic neuropathy and painful diabetic neuropathy.

Alise Kalteniece[1], Maryam Ferdousi[1], Ioannis Petropoulos[2], Shazli Azmi[1], Safwaan Adam[1], Hassan Fadavi[1], Andrew Marshall[1], Andrew JM Boulton[1], Nathan Efron[3], Catharina G Faber[4], Giuseppe Lauria[5], Handrean Soran[1], Rayaz A Malik [1,2,*]

[1] Institute of Cardiovascular Sciences, Cardiac Centre, Faculty of Medical and Human Sciences, University of Manchester and NIHR/Wellcome Trust Clinical Research Facility, Manchester, UK.

[2] Weill Cornell Medicine-Qatar, Research Division, Qatar Foundation, Education City, Doha, Qatar.

[3] Queensland University of Technology, School of Optometry and Vision Science, Brisbane, Australia.

[4] Department of Neurology, School of Mental Health and Neuroscience, Maastricht University Medical Center, Maastricht, The Netherlands.

[5] Neuroalgology Unit and Skin Biopsy, Peripheral Neuropathy and Neuropathic Pain Center, IRCCS Foundation "Carlo Besta" Neurological Institute, Milan, Italy.

Corresponding author:

*E-mail: ram2045@qatar-med.cornell.edu & rayaz.a.malik@manchester.ac.uk

Supplementary table S1

	T1DM (n=52)	T2DM (n=64)
**Age**	48.53 ± 2.16 *	62.02 ± 1.19
**Duration of diabetes**	27.4 ± 2.39*	11.07 ± 1.08
**HbA1c (mmol/mol)**	7.77 ± 0.22^	7.06 ± 0.19
**CNFD (no./mm^2)£**	25.67 ± 1.27	24.7 ± 0.96
**CNBD (no./mm^2)£**	57.91 ± 4.37	61.84 ± 4.11
**CNFL (mm/mm^2)£**	20.79 ± 0.92	23.68 ± 0.89
**IWL (mm/mm^2)£**	22.17 ± 1.39	23.19 ± 1.12
**ANFL (mm/mm^2)£**	21.48 ± 1.03	23.44 ± 0.9
**TNFL (mm/mm^2)£**	42.96 ± 2.06	46.88 ± 1.8

CCM parameters in patients with Type 1 (T1DM) and Type 2 (T2DM) diabetes mellitus. £ represents values adjusted for age, duration of diabetes and HbA1c using ANCOVA; *P<0.0001 compared to T2DM, ^P<0.05 compared to T2DM
